# Molecular dynamics simulations of the self-organization of side-chain decorated polyaromatic conjugation molecules: phase separated lamellar and columnar structures and dispersion behaviors in toluene solvent†

**DOI:** 10.1039/c7ra13101a

**Published:** 2018-03-21

**Authors:** Lanyan He, Pingmei Wang, Lipeng He, Zhou Qu, Jianhui Luo, Baoliang Peng, Xianqiong Tang, Yong Pei

**Affiliations:** Key Laboratory for Green Organic Synthesis and Application of Hunan Province, Key Laboratory of Environmentally Friendly Chemistry and Applications of Ministry of Education, Xiangtan University Hunan Province 411105 China ypnku78@gmail.com; Department of Civil Engineering and Mechanics, Xiangtan University Xiangtan 411105 China tangxq85@xtu.edu.cn; Research Institute of Petroleum Exploration & Development (RIPED), PetroChina Beijing 100083 China luojh@petrochina.com.cn; Key Laboratory of Nano Chemistry (KLNC), CNPC Beijing 100083 China

## Abstract

The self-organization of five model side-chain decorated polyaromatic asphaltene molecules with or without toluene solvent was investigated by means of atomistic molecular dynamic (MD) simulations. It was found that the organizational structure of polycyclic asphaltene molecules is significantly affected by the position and length of side chains. In the present study, two types of phase-separated stacking configurations, including the phase separated lamellar structure (PSLS) and the phase separated columnar structure (PSCS), were found. The PSLS and PSCS were also maintained in the presence of a small amount of toluene additive (30% wt fraction). When adding excess toluene molecules, the asphaltene molecules formed highly dispersed nanoaggregates. The dynamic properties of the π–π stacking structures in the PSLS and PSCS, as well as the nanoaggregates, were probed. It was found that the number and size of alkyl side chains significantly impacted the size and number of π–π stacking structures in the aggregates. Through tracking the structural evolution of the nanoaggregates, a possible dissociation mechanism of nanoaggregates is also suggested.

## Abstract 2

The self-organization of five model side-chain decorated polyaromatic asphaltene molecules with or without toluene solvent was investigated by means of molecular dynamic (MD) simulations.

## Graphical abstract

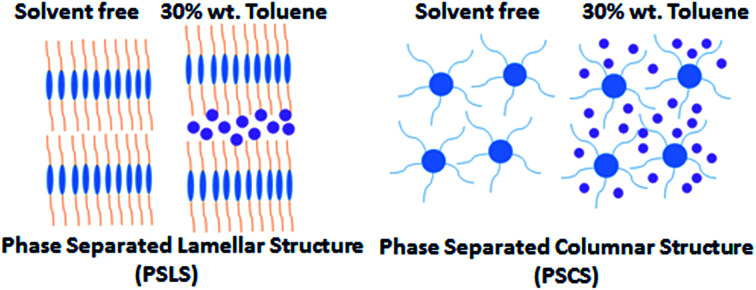

## 1. Introduction

Asphaltenes are the most complex component lying in crude oils and are composed of aliphatic chains, polyaromatic condensed rings, organometallic complexes, and heteroatoms such as N, O, and S.^1,2^ On the basis of the concept of solubility, asphaltenes are generally defined as the part of petroleum that is insoluble in low molecular *n*-alkanes (*n*-pentane and *n*-heptane) but soluble in aromatic solvents, such as toluene or benzene.^3^ Asphaltenes have a strong tendency to self-associate, and it was discovered that when the concentration of asphaltenes in a solvent such as toluene exceeds the critical nanoaggregate concentration (CNAC), aggregation phenomena can occur.^4^

The aggregation and precipitation behavior of asphaltenes has been previously studied by several experimental methods, such as small-angle X-ray scattering,^5^ small-angle neutron scattering,^6,7^ and vapor pressure osmometry.^6,8^ Due to the complex nature of asphaltene molecules, the mechanism of aggregation-dispersion and the interaction between asphaltene molecules are not clear, and the microscopic details of the aggregation process and the driving forces still require further examination.

Recently, based on the earlier proposed Yen model, Mullins *et al.*^9–11^ have proposed a modified Yen model (called the Yen–Mullins model). In this model, when the concentration of asphaltene reaches the critical value (CNAC, 50–150 mg L^−1^), the asphaltene molecules form nanoaggregates with an aggregation number generally less than 10. With increasing concentration, these nanoaggregates form clusters of aggregates. Rogel *et al.*^12^ pointed out that the primary factor that promoted the association of asphaltene molecules was the van der Waals interaction, with the effect of electrostatic interactions and conformation changes being relatively smaller. Takanohashi *et al.*^13^ studied the stability of asphaltene aggregates at different temperatures using molecular mechanics (MM) and molecular dynamics (MD) simulations. They concluded that the hydrogen bond between asphaltene molecules dissociated at 523 K, while the π–π interactions appeared to be stable. Murgich *et al.*^14^ performed molecular mechanics calculations for large continental type asphaltene molecules with 24 aromatic rings and showed that asphaltene aggregation was mainly driven by the stacking of the polyaromatic cores. They confirmed that the π–π interaction was the dominant role of asphaltene aggregation. Pacheco-Sánchez *et al.*^15^ optimized the geometrical structure of four molecular models (the original structures were proposed by Groenzin and Mullins *et al.*,^16^ Speight,^17^ Zajac *et al.*^18^ and Murgich *et al.*^14^) and found that the asphaltene aggregation structure is consistent with the Yen model. The simulation results indicated that in addition to face-to-face stacking, molecular orientations between asphaltene molecules, π-offset, and T-shaped stacking were also observed and showed good agreement with the results of Leach *et al.*^19^ Jian *et al.*^20^ investigated the effect of aliphatic side-chain length on the aggregation behavior of asphaltene in aqueous solution by MD simulations. They discovered that there was a non-monotonic relationship between the extent of aggregation and the side-chain length, and asphaltene molecules with very short or very long side chains can form dense aggregates. Rogel *et al.*^21^ demonstrated the dimer formation of aggregates in pure *n*-heptane, but not in a toluene/*n*-heptane mixture or in pure toluene. There is a greater decrease of the interaction energy between the asphaltenes at the solvent, indicating a weakness of the asphaltene–asphaltene association in this solvent. Ungerer *et al.*^22^ found that the aggregation of the continental asphaltene model was irreversible by MD simulations, and the asphaltene aggregation in toluene was weaker than that in *n*-heptane. Buenrostro-Gonzalez *et al.*^23^ demonstrated that a relationship with π-bond stacking and the alkane steric of stacking was critical for maintaining the solubility of the asphaltenes in toluene, and this hypothesis was explored by experimental techniques such as fluorescence depolarization and fluorescence emission spectroscopy.

Because the aggregation behavior of asphaltene is closely related to its molecular structure, it is necessary to study the relationship between asphaltenes from the atomic and molecular level. In this work, we studied the self-organization behaviors of a series of model alkyl side chain-decorated polyaromatic molecules by means of molecular dynamics simulations. By adjusting the number, position, and length of aliphatic side chains surrounding a condensed polycyclic aromatic nucleus, a series of model polyaromatic asphaltene molecules was constructed. From studying the self-organization behaviors of these model asphaltene molecules, the driving forces of aggregation and the molecular structure effects on the aggregate structures were analyzed. The aggregation and dispersion behaviors of these model asphaltene molecules in the toluene solvent or with toluene additive (30% weight fraction) were studied as well.

## 2. Simulation model and methods

### 2.1 Building model asphaltene molecular structures

It is generally accepted that asphaltene molecules can be represented as two primary types: ‘continental’ and ‘archipelago’.^24,25^ The ‘continental’ model consists of a large polycyclic aromatic core bonded by aliphatic side chains and cycloalkane rings, whereas the ‘archipelago’ model consists of an aromatic group connected by aliphatic chains and thiol ethers.

**Fig. 1:**
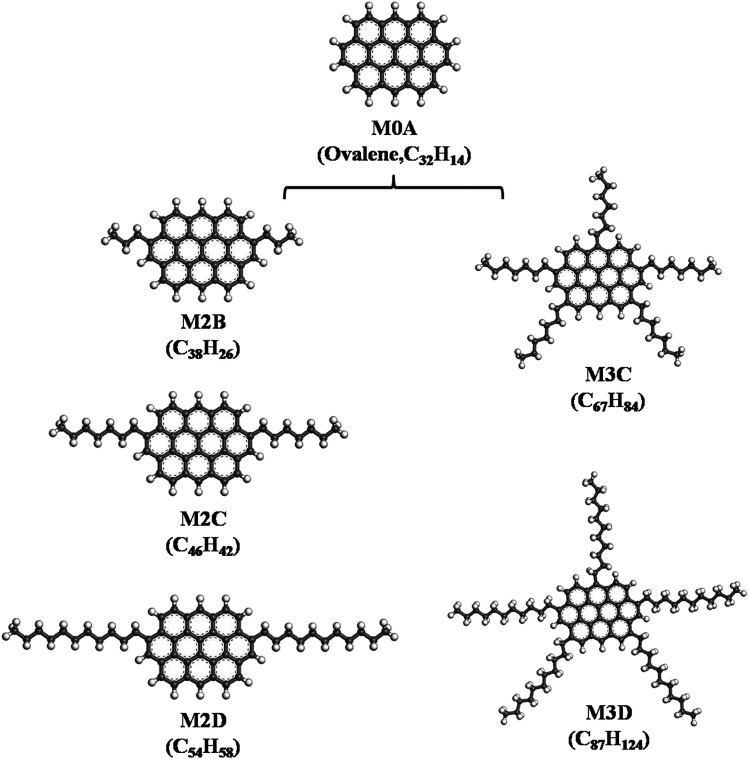
Five types of side-chain decorated model ‘continental’ type asphaltene molecules constructed from the mother M0A molecule (ovalene), labeled M2B, M2C, M2D, and M3C, M3D, respectively.

In this work, we constructed asphaltene molecules based on the ‘continental’ model. As shown in Fig. 1, five ‘continental’ type model asphaltene molecules were constructed starting from a mother structure M0A (ovalene), which is composed of ten fused phenyl rings. Starting from M0A: (I) by adding a certain number of aliphatic side chains, the M2B, M2C, and M2D containing two aliphatic side chains with different lengths and the M3C and M3D containing five aliphatic side chains with different lengths are constructed. From designing these molecular structures, the effects of length and number of aliphatic side chains on the association behavior of model asphaltene molecules were explored. The five model molecules have the H/C ratio in a range of 0.95–1.42, which is close to the average compositions of asphaltene (the H/C ratio is 0.9–1.3) quoted by Rogel *et al.*^12^

### 2.2 Molecular dynamic simulations

All MD simulations were performed with the large-scale atomic/molecular massively parallel simulator (LAMMPS) program package.^26^ The time step was set as 1 fs. The intra- and inter-molecular interactions were described with the polymer consistent force field (PCFF),^27^ which is suitable and reliably used for describing alkyl chains, molecular clusters, and polymers. The long-range electrostatic interactions were calculated using the particle–particle–particle–mesh (PPPM) method. Periodic boundary conditions were employed in all simulations, and the snapshots of molecular trajectories were visualized by Visual Molecular Dynamics (VMD) software.^28^ The velocity Verlet algorithm was used for integration in all the MD simulations.

Two types of simulation models were built to investigate the association behaviors of model asphaltene molecules, *e.g.*, solvent-free, and mixed asphaltene and toluene solvent molecular models. For the latter model, we have considered two types of models: (i) toluene as an additive (30% wt fraction) and (ii) toluene as the solvent. The number of toluene and asphaltene molecules used in these simulation models is displayed in Table 1.

**Table 1:** The number of toluene and asphaltene molecules used in different types of simulation models. The asphaltene molecules involved in these simulation systems are M2B, M2C, M2D, M3C, and M3D displayed in Fig. 1. Note: ^a,b,c,d,e^ represent M2B, M2C, M2D, M3C, and M3D systems, respectively

Simulation models	Number of asphaltene molecules	Number of toluene molecules	Temperature/pressure
Pure asphaltene molecular model	60	0	533 K/1 atm
Asphaltene/toluene (30% wt fraction)	60	135^a^/166^b^/197^c^/248^d^/326^e^	300 K/1 atm
Asphaltene/toluene (excess)	30	2900	300 K/1 atm

For the solvent-free system, the simulation process included four steps: (I) in the initial simulation box, 60 asphaltene molecules were randomly distributed, and the initial density of the system was set to 0.6 g cm^−3^, which was lower than the ordinary density of asphaltene. (II) Energy minimization was performed for each system to obtain the optimal configuration of molecules (including bond length, angles, *etc.*). (III) After energy minimization, each system was equilibrated by a 30 ns isobaric–isothermal (NPT) ensemble simulation at a constant pressure of 1 bar, and the temperature was maintained at 533 K. During the equilibrium period, the density and energies (including the kinetic energy and the potential energy) were monitored to ensure that the system reached the equilibrium state. (IV) The production stage was examined. After the system was fully relaxed and reached the equilibrium state by NPT MD simulations, a series of long-time (150–250 ns) canonical ensemble (NVT) simulations was performed, and the atomic position, force, velocity, *etc.*, were collected for later analysis.

For the mixed asphaltene and toluene molecular system, the simulation process included the following steps: (I) in the initial simulation box, asphaltene and toluene molecules were initially randomly distributed in a cubic simulation box, and the initial density of the system was set to 0.6 g cm^−3^. (II) Energy minimization was then performed for each system to obtain the optimal configuration of molecules. (III) After energy minimization, each system was equilibrated by an isobaric–isothermal (NPT) ensemble simulation at a constant pressure of 1 bar, and the temperature was maintained at 300 K. During the equilibrium period, the density and energies (including the kinetic energy and the potential energy) were monitored to ensure that the system reached the equilibrium state. (IV) The production stage was examined. After the system was fully relaxed and reached the equilibrium state, the atomic position, force, velocity, *etc.* were collected for later analysis.

## 3. Results and discussion

### 3.1 Self-association behavior of side chain-decorated polyaromatic asphaltene molecules

Pacheco-Sánchez *et al.*^15^ performed MD simulations on several asphaltene molecular models in a vacuum and demonstrated that the face-to-face stacking is not the only possible direction, as asphaltenes molecules also form π-offset stacking and T-shaped geometries. Murgich *et al.*^29^ suggested that the interlayer spacing of aliphatic side chain branched asphaltene molecules was greater than that of unbranched asphaltenes. Zhang *et al.*^30^ studied the orientation and structure of the molecules in the two ternary asphalt mixtures, and one type of asphaltene molecule had a long alkane side chain, and the other one was composed of many fused polycyclic aromatic rings with very short chains. All these previous studies have suggested that the presence of aliphatic side chains had a remarkable effect on the aggregational structure of asphaltene molecules.

Herein, the effect of branched aliphatic side chains on the self-assembly behavior of ‘continental’ asphaltene molecules was studied by simulating five model molecular systems with identical aromatic nuclei but different number and length alkyl side chains. Firstly, we performed a series of MD simulations based on the M2B, M2C, and M2D molecular systems at 533 K to investigate the effect of length of side chains on the self-association properties. In these model molecular systems, two alkyl side chains containing three, seven, and eleven carbon atoms were symmetrically added to a parental M0A molecule (seen in Fig. 1). From 30 ns pre-equilibrium NPT and over 250 ns NVT MD simulations, self-association behavior is clearly found within these molecular systems.

**Fig. 2:**
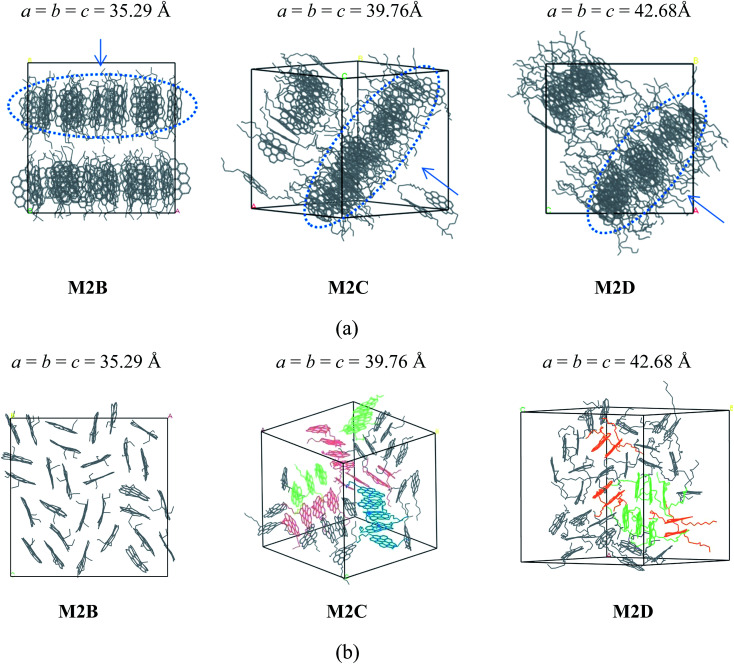
(a) Snapshot of the equilibrated molecular configurations of three polyaromatic ‘continental’ type asphaltenes M2B, M2C, and M2D (with different length alkyl side chains). The molecular dimer, trimer, tetramer, and pentamer aggregation structures are colored by orange, green, red, and blue, respectively. H atoms were hidden for clarity. The PSLS packing structures are seen. (b) Comparison of M2B, M2C, and M2D molecular aggregate configurations in a single molecular monolayer (circled with dashed blue lines in a). The H atoms were hidden for clarity. The values *a*, *b*, and *c* are the cell parameters of the simulation box, in units of Angstroms. In (a), the arrow denotes the view direction of the layer packing structures as displayed in (b).

As shown in Fig. 2a, the M2B, M2C, and M2D molecular systems containing two branched alkyl side-chains show interesting self-association behaviors. In the initial stage of MD simulations, molecules are fully randomly distributed in the simulation box. As the MD simulations progress, the molecules first rapidly formed smaller aggregates caused by the inter-molecular π–π interactions, and after a few ns MD simulations (see the details in Figure S1–S3 in the ESI†), these smaller aggregates finally formed layered structures. As seen in the MD snapshots displayed in Fig. 2a, the final equilibrium molecular packing structures of M2B, M2C, and M2D formed unique ‘phase-separated lamellar structure’ (PSLS), in which asphaltene molecules aggregated into some lamellar monolayers, and these lamellar monolayers were separated with the alkyl chains.

We note that for the side chain-free ovalene molecule (M0A), single X-ray diffraction indicated herringbone molecular packing structures,^31,32^ and altered molecular packing structures were formed. At present, the observation of PSLS in the M2B, M2C, and M2D molecular systems implied that the alkyl side chains interfered with the interactions between the aromatic cores, and the long-range ordered face-to-face π–π stacking structure was not found. Experimental studies^33,34^ also showed that the alkane chains introduced steric interference to the π–π interaction of aromatic cores and disrupted the stacking of polyaromatic cores in the ‘continental’ type of asphaltenes, which is consistent with our simulation results. However, it was found that the side chains of asphaltene molecules were distributed in the periphery of the π–π stacking structure. In other words, the side chains were not inserted into the space between the aromatic cores. These uniformly distributed alkyl side chains may be a primary reason for the formation of PSLS.

Furthermore, the time required to achieve stable PSLS of M2B, M2C, and M2D systems from the initial fully random structure was different. Generally speaking, the longer the alkyl side chains, the longer the time that was required to reach the equilibrium configuration. For the M2B system, the PSLS is formed after an approximately 4.5 ns simulation. For comparison, the formation of PSLS in the M2C and M2D requires more time due to the longer side chain lengths (approximately 7 and 11 ns, respectively).

**Fig. 3:**
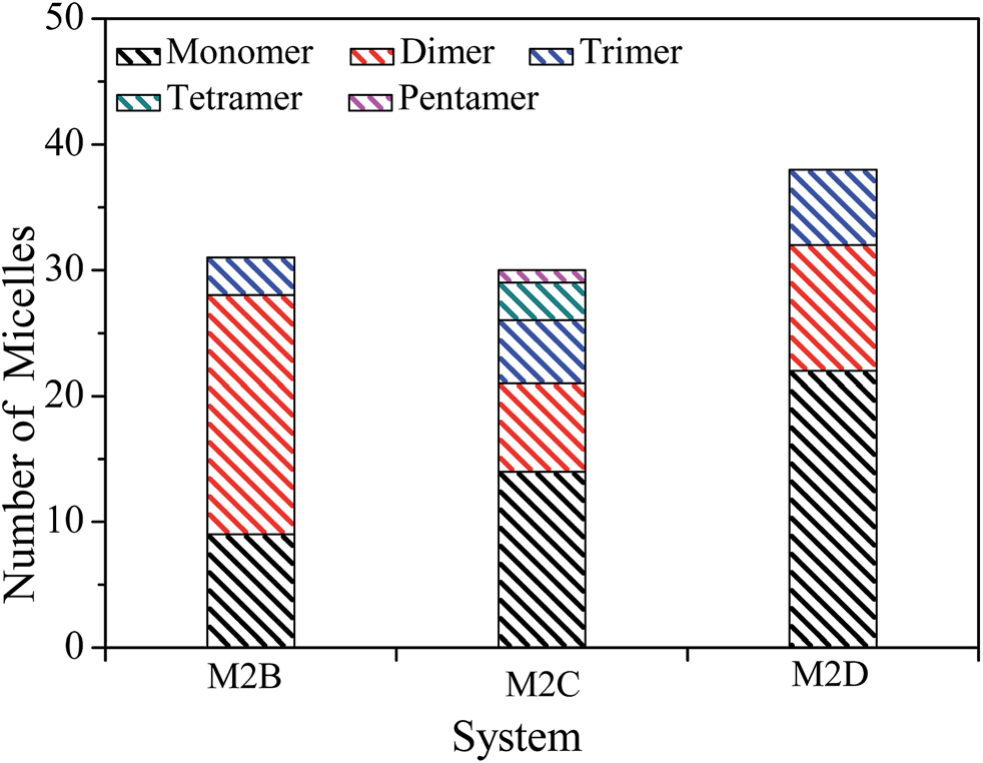
The number of molecular monomers, face-to-face π–π dimers, trimers, tetramers, and pentamers within the M2B, M2C, and M2D molecular systems.

The spatial orientation of M2B, M2C, and M2D molecules in the PSLS are diversified, including face-to-face, offset stacking, and T-type stacking. Additionally, the aggregation structures of M2B, M2C, and M2D molecules were generally smaller molecular aggregates such as dimers and trimers, and the long-range ordered face-to-face π–π packing structures were not seen. In Fig. 3, the number of the face-to-face π–π stacking molecular aggregates was counted, and it could be seen that with the increase of length of the alkyl side-chain from three to eleven carbon atoms, the aggregated extent of three systems initially increased and then decreased afterwards. The M2C molecular system owns the largest degree of face-to-face π–π stacking.

**Fig. 4:**
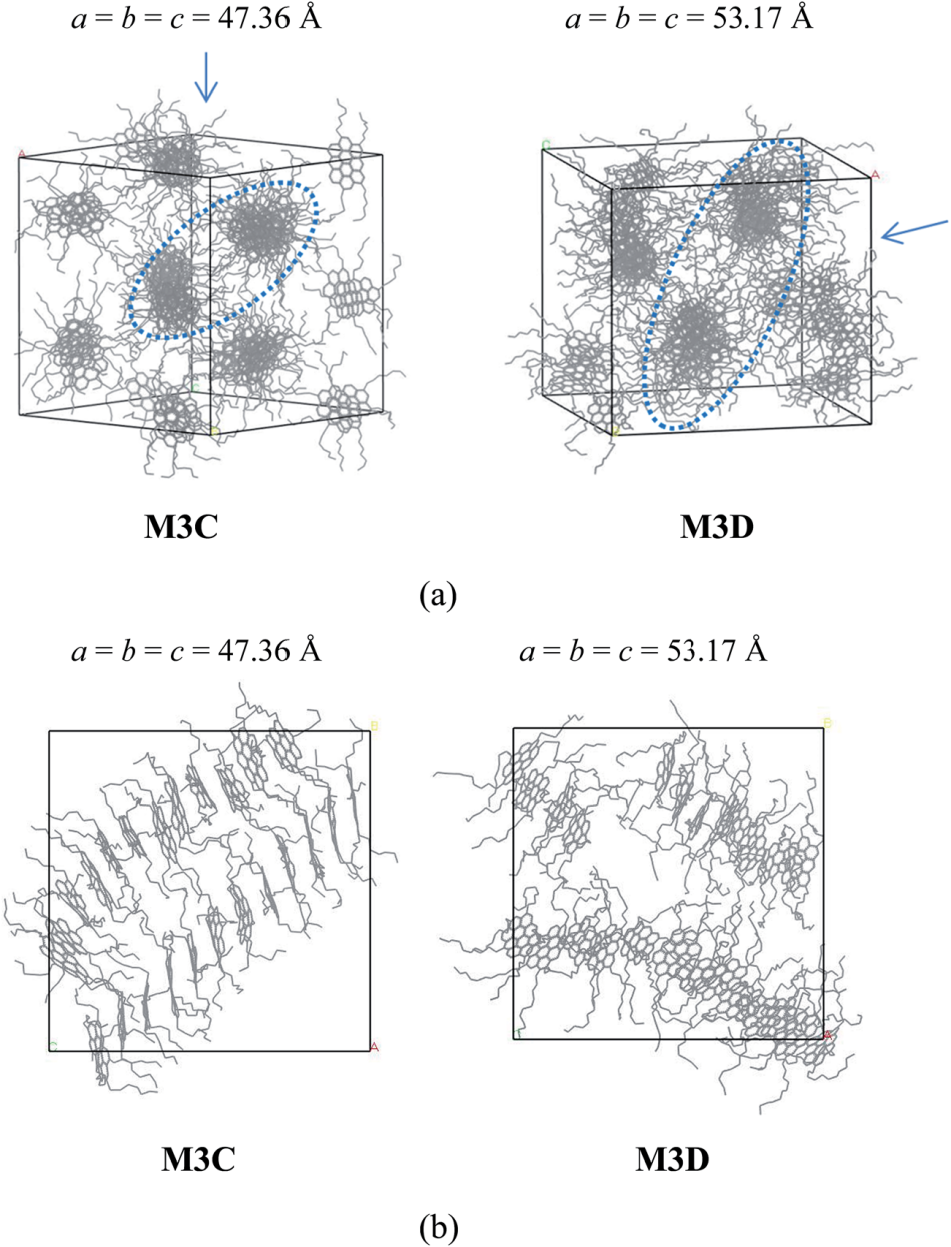
(a) Snapshots of the equilibrium molecular configuration of the M3C and M3D systems. ‘Phase separation columnar structure’ (PSCS) was found for both systems. (b) Morphology of rod-like aggregates formed by M3C and M3D molecules. H atoms were hidden for clarity. In (a), the arrow denotes the view direction of the columnar packing structures, as shown in (b). The values *a*, *b*, and *c* are the cell parameters of the simulation box, in units of Angstroms.

The molecular aggregation behavior of two model alphaltene molecules labeled by M3C and M3D was investigated. In these two model molecules, the number of alkyl side chains was increased to five, and the length of alkyl chains in M3C and M3D was found to be identical to that of the M2C and M2D system, respectively. By simulating the self-assembly behaviors of the M3C and M3D molecular systems, the effect of highly branched alkyl side chains on the association behaviors of polyaromatic asphaltene was investigated. From the equilibrated configuration of the M3C and M3D systems as shown in Fig. 4, it can be observed that the polyaromatic asphaltene molecules with a higher degree of side chain branching (M3C and M3D) formed a series of ordered ‘columnar’ stacking structures.

At the initial stage of simulations, the disordered asphaltene molecules first formed smaller face-to-face π–π stacking structures with random orientations, and then these randomly distributed smaller stacked aggregates gradually combined into a series of ‘columnar’-like stacking structures (Fig. S4 and S5†). By investigating the equilibrium molecular configurations in the M3C and M3D systems, it was found that within each ‘columnar’-like stacking structures, the aromatic nucleus adopted face-to-face π–π stacking, and these ‘columnar’-like stacking structures were separated by the alkyl side chains, showing a novel ‘phase separation columnar structure’ (PSCS).

The length of the side chains affected the PSCS. As shown in Fig. 4, in the M3C molecular system, due to the relatively shorter alkyl side chains, the ‘columnar’ like stacking structures were very ordered and nearly parallel to each other. However, when the length of the alkyl chains increased, the ‘columnar’-like aggregates showed disordered relative orientations. We noted that the molecules stacked on top of each other, and the columnar arrangement as observed in the M3B and M3C systems was similar to that of discotic liquid crystals. Most discotic thermotropic liquid crystals are flat molecules with a central aromatic core and several aliphatic chains attached at the edges. Our present MD simulation results agree with previous experimental and theoretical reports describing the packing structure of hexabenzocoronene derivates.^35^

**Fig. 5:**
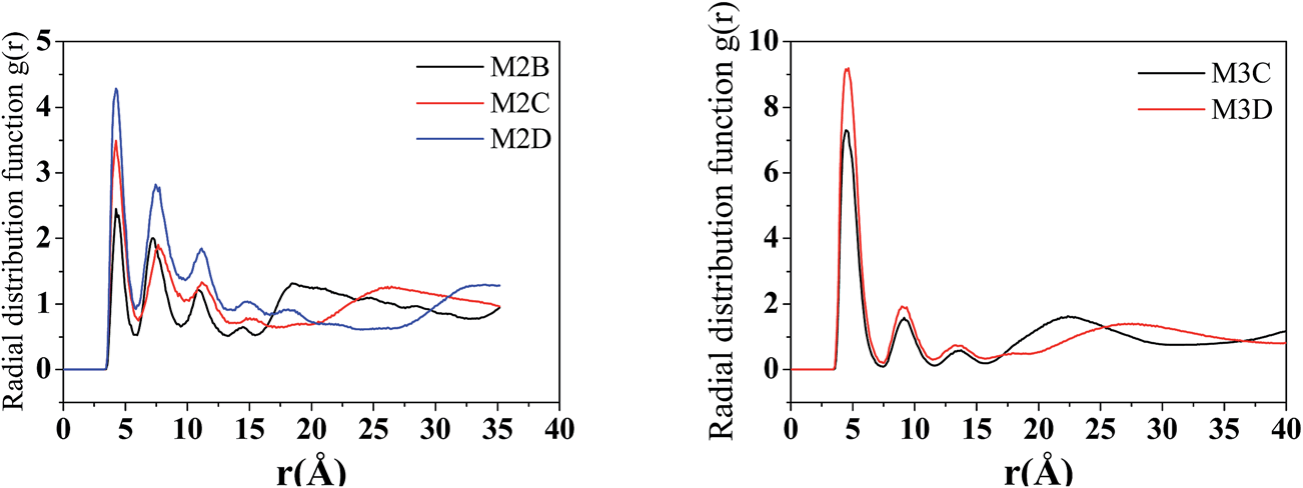
The radical distribution function *g*(*r*) of asphaltene–asphaltene molecules in five molecular aggregation structures (*r* is the distance between the centers of the aromatic cores).

The radical distribution function *g*(*r*) of mass centers of aromatic cores of five types of model systems was calculated and is displayed in Fig. 5. As seen from Fig. 5, for all five systems, a strong peak is seen at approximately 4.6 Å. This peak distance is greater than the π–π stacking distance of approximately 3.5 Å. We noted that the present calculation of *g*(*r*) considers the aromatic core center–center distance rather than the vertical distance between the aromatic core planes. It can be considered that if the mass center distance between two aromatic cores is less than approximately 5 Å, two asphaltene molecules will form face-to-face π–π stacking. However, for the M2B, M2C, and M2D with the same number of alkyl side chains, the height of first *g*(*r*) peak shows an order of M2D > M2B > M2C, which indicates that with the increase of the side chain length, larger-sized π–π stacking structures are formed. For the M3C and M3D systems, a dominant *g*(*r*) peak is also seen at the position of approximately 4.6 Å. In comparison to the M2B, M2C, and M2D systems, the *g*(*r*) peaks above 5 Å are relative weaker. This indicates that the M3C and M3D formed more compact π–π stacking structures than those of M2B, M2C, and M2D molecules.

### 3.2 Association of side chain-decorated polyaromatic asphaltene molecules in toluene solvents

When soluble in a crude or aromatic solvent, the aggregation behaviors of asphaltene molecules may be quite different from the behaviors exhibited during their bulk phase. The asphaltenes are known to aggregate in the nanometer length-scale, forming “nanoaggregates” in solvents such as toluene.^36–41^

In this part of the discussion, the assembly and aggregation behaviors of five types of model asphaltene molecules in the presence of toluene solvents are explored. We have considered toluene as an additive (30% wt fraction) and toluene as a solvent (*cf.*Table 1). In the first simulation system, 30% weight fraction toluene molecules are mixed with the model asphaltene molecules. To the best of our knowledge, few studies have investigated the assembly behaviors of asphaltene molecules with a small amount of toluene additives.

**Fig. 6:**
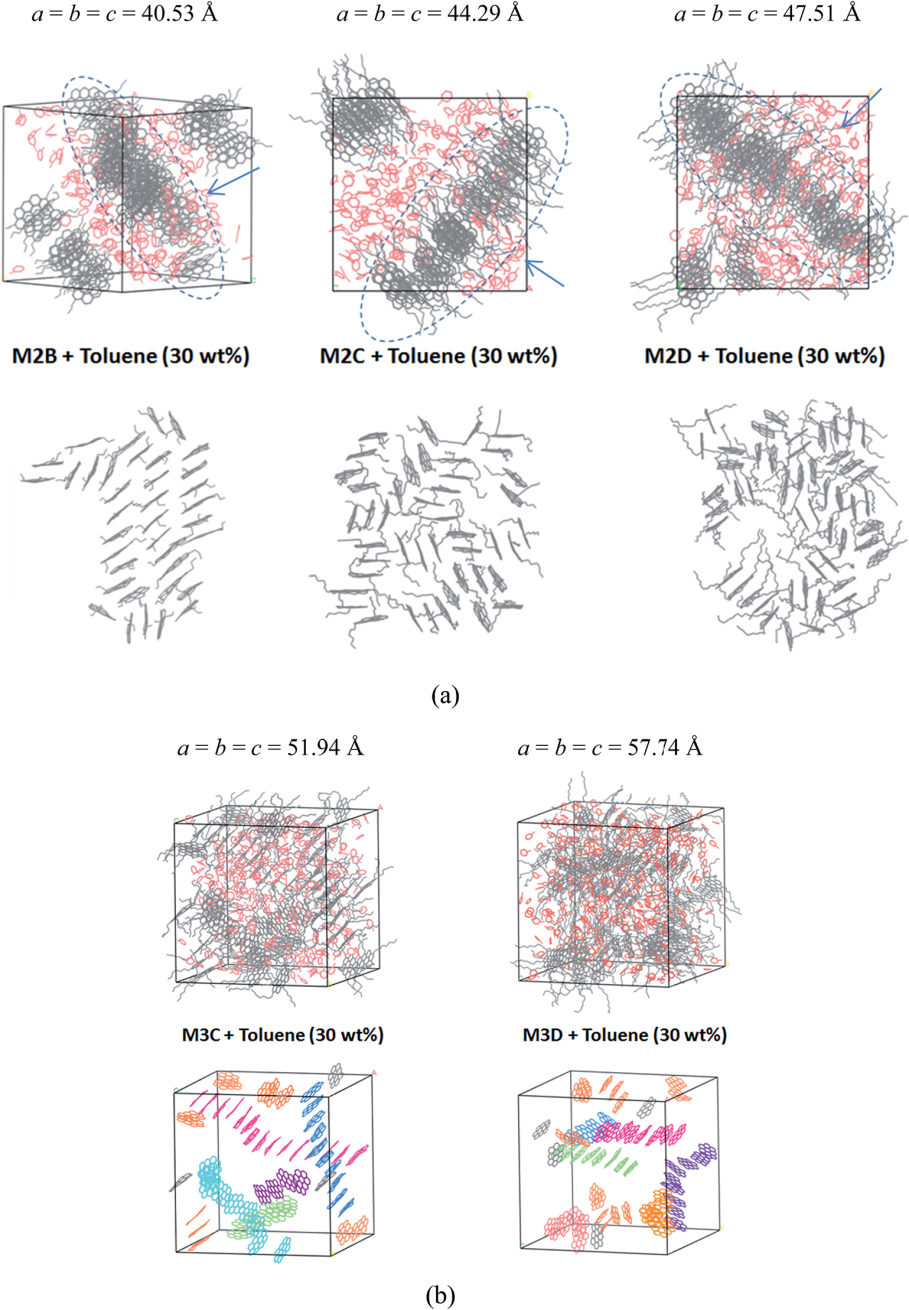
Molecular snapshots of the equilibrated configuration of polyaromatic asphaltene molecules (a) M2B, M2C, and M2D with 30% wt fraction toluene molecules and (b) M3C and M3D and toluene molecules. In order to discern the packing structure of the aromatic nucleus within the M3C and M3D molecules, the alkyl side chains and H atoms are not displayed. Toluene molecules are highlighted in red. The values *a*, *b*, and *c* are the cell parameters of the simulation box, in units of Angstroms. In (a), the arrow denotes the view direction of the columnar packing structures.

Fig. 6a displays the equilibrium configuration and snapshots of MD trajectories of mixed asphaltene and toluene (30% wt fraction) molecular systems. Interestingly, for all types of systems, it can be found that the presence of toluene additives does not significantly affect the aggregation structure of asphaltene molecules. The molecular snapshots displayed in Figure S6 and S7† clearly indicate the phase separation process in all systems. In the initial simulation boxes, asphaltene and toluene molecules are randomly distributed, and some toluene molecules are distributed in the spaces among the aromatic nucleus. As the simulations progress, the toluene molecules are rapidly extruded from the space between the aromatic nucleus, and the asphaltene molecules gradually self-assemble into π–π stacking aggregates.

In the M2B, M2C, and M2D systems, the PSLS is formed. The toluene molecules are fully extruded from the space between the aromatic cores and form an additive layer between the asphaltene molecular layers. However, the effect of toluene molecules can be still observed in that the dense layer aggregates were penetrated by the toluene molecules. In the M2B, M2C, M2D systems, some toluene molecules have penetrated into the lamellar layers, as shown in Fig. 6a. However, due to the smaller amount of solvent, the isolated nanoaggregates are not seen.

For the M3C and M3D molecular systems, as shown in Fig. 6b, columnar stacking molecular aggregates are formed after the system reaches the equilibrium state. However, unlike the solvent-free system, the columnar aggregates are significantly dispersed by the toluene additives. In both systems, as shown in Fig. 6b, we have identified some long rod-like π–π stacking structures that are separated by toluene molecules.

**Scheme 1:**
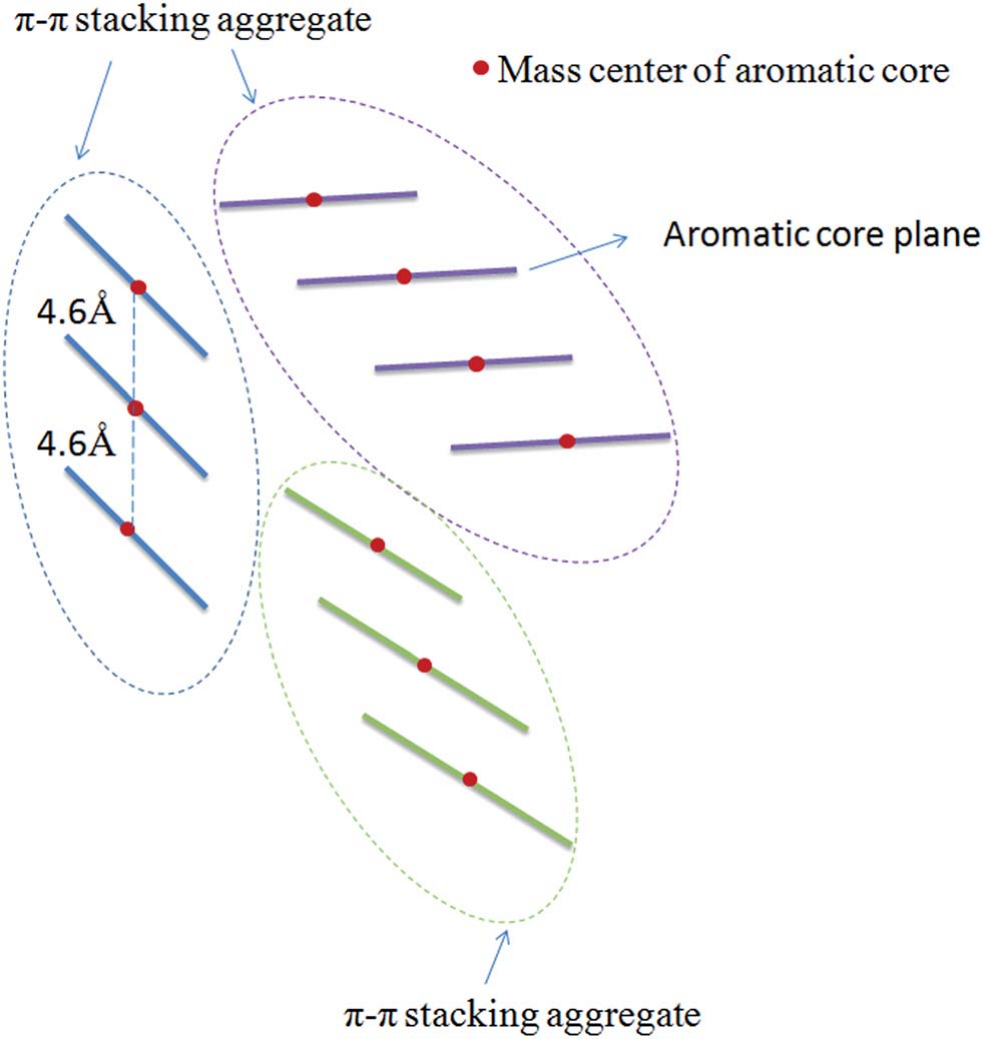
Schematic definition of the π–π stacking molecular aggregates. From the *g*(*r*) curves, the distance between the mass centers of two nearby π–π stacking aromatic cores is approximately 4.6 Å. Here, we used a slightly larger value (6.0 Å) to count the number of π–π stacking molecular aggregates.

In order to track the dynamic behaviors of asphaltene molecules in the PSLS and PSCS, we counted the size (only the maximum size aggregate is counted) and number of molecular aggregates of five systems at different MD simulation stages. We defined the size of asphaltene aggregate according to the mass center distances of the aromatic nucleus. As shown in Scheme 1, we consider the nearby asphaltene molecules with a mass center distance less than 6 Å (slightly larger than the position of the first *g*(*r*) peak, approximately 4.6 Å in Fig. S8†) to belong to one π–π stacking aggregate.

**Fig. 7:**
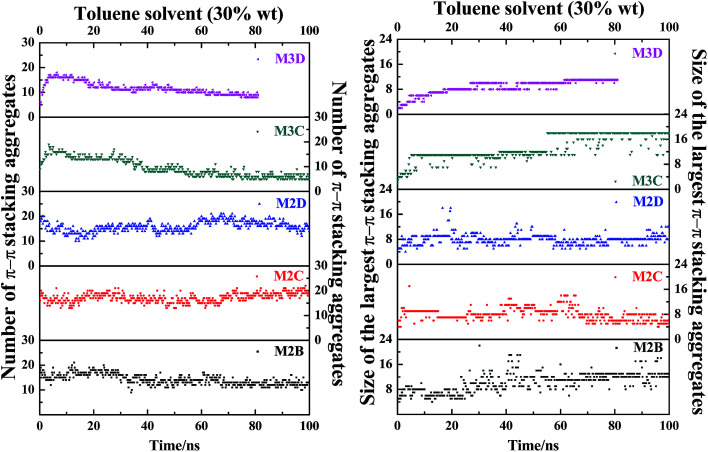
The evolution of size and number of π–π stacking molecular aggregates in five systems (M2B, M2C, M2D, M3C, and M3D, with 30% wt toluene solvent that was mixed with the asphaltene molecules) along with the time of MD simulations. The size of the largest π–π stacking molecular aggregates is defined by the number of asphaltene molecules within the largest-sized aggregates.

Fig. 7 plots the evolution of the size and number of π–π stacking molecular aggregates in five systems along with the time of MD simulations. From Fig. 7, in all systems, the size and number of π–π stacking molecular aggregates in PSLS and PSCS changed rapidly with the simulation time. This indicates that the π–π stacking molecular aggregates within the PSLS and PSCS underwent rapid deformations and reformations. This result agrees with the previous theoretical studies of nanoaggregation of asphaltene structures in the toluene and heptanes.^39–41^ It was observed that in the solvent, the asphaltenes form dimers and trimers, and the formed dimers and trimers can separate and reform other nanoaggregates with other asphaltene molecules.

Herein, for the M2C and M2D plus toluene molecular systems, the number of π–π stacking molecular aggregates within PSLS does not show significant fluctuation within 100 ns simulations, and the largest π–π stacking aggregate size (defined by the number of asphaltene molecules within the molecular aggregates) is generally less than 8. However, in the M2B molecular system, larger-sized π–π stacking molecular aggregates containing 13–14 molecules were found. The difference in π–π stacking aggregate sizes can be also seen from the snapshot displayed in Fig. 6a. From Fig. 6a, in the M2B molecular systems, due to the penetration of the solvent molecules, a large cavity is formed in the lamellar packing structure. The M2B molecules may adjust their orientations more easily and form more compact π–π stacking structures.

For comparison, the M3C and M3D containing five alkyl side chains show much different dynamic behaviors in the PSCS. From Fig. 7, at the initial simulation stage, a rapid increase in π–π stacking aggregate size is seen in both M3C and M3D molecular systems. When the systems reached the equilibrium states, some stable large-sized aggregates formed within the PSCS of the M3C and M3D systems. For the M3C system, the size of the largest aggregate even exceeds 16, and the aggregate remained stable over a long period of time. However, the size of the π–π stacking aggregates in the M3D system is much smaller than that of M3C molecules. The largest π–π stacking aggregate involves no more than 11 molecules. The observation of much different size and number of π–π stacking molecular aggregates in the PSLS and PSCS indicates that the length and distribution of alkyl side chains significantly impacts the association properties of polyaromatic alphaltene molecules.

Finally, the dispersion and aggregation behaviors of the side chain-decorated polyaromatic asphaltene molecules in the excess toluene solvent were investigated. Unlike the above models, the presence of excess solvent molecules entails more freedom for the asphaltene molecules. The settings of five simulation systems are displayed in Table 1, in which 30 asphaltene molecules are solvated in 2900 toluene molecules (the weight fraction of asphaltene molecule ranges from 5.1–11.6%).

In agreement with previous studies,^39–41^ the asphaltene molecules have fully dispersed and formed ultrasmall-sized nanoaggregates in the toluene solvent. Herein, we focus on the effect of aliphatic side chains on the size and aggregation behaviors of nanoaggregates. The aromatic core center–center radical distribution function *g*(*r*) is first calculated (Fig. S9†). The *g*(*r*) of five asphaltene systems show a common dominant peak positioned at approximately 4.6 Å, which indicates that the π–π stacking is the primary association configuration of the nanoaggregates.

**Fig. 8:**
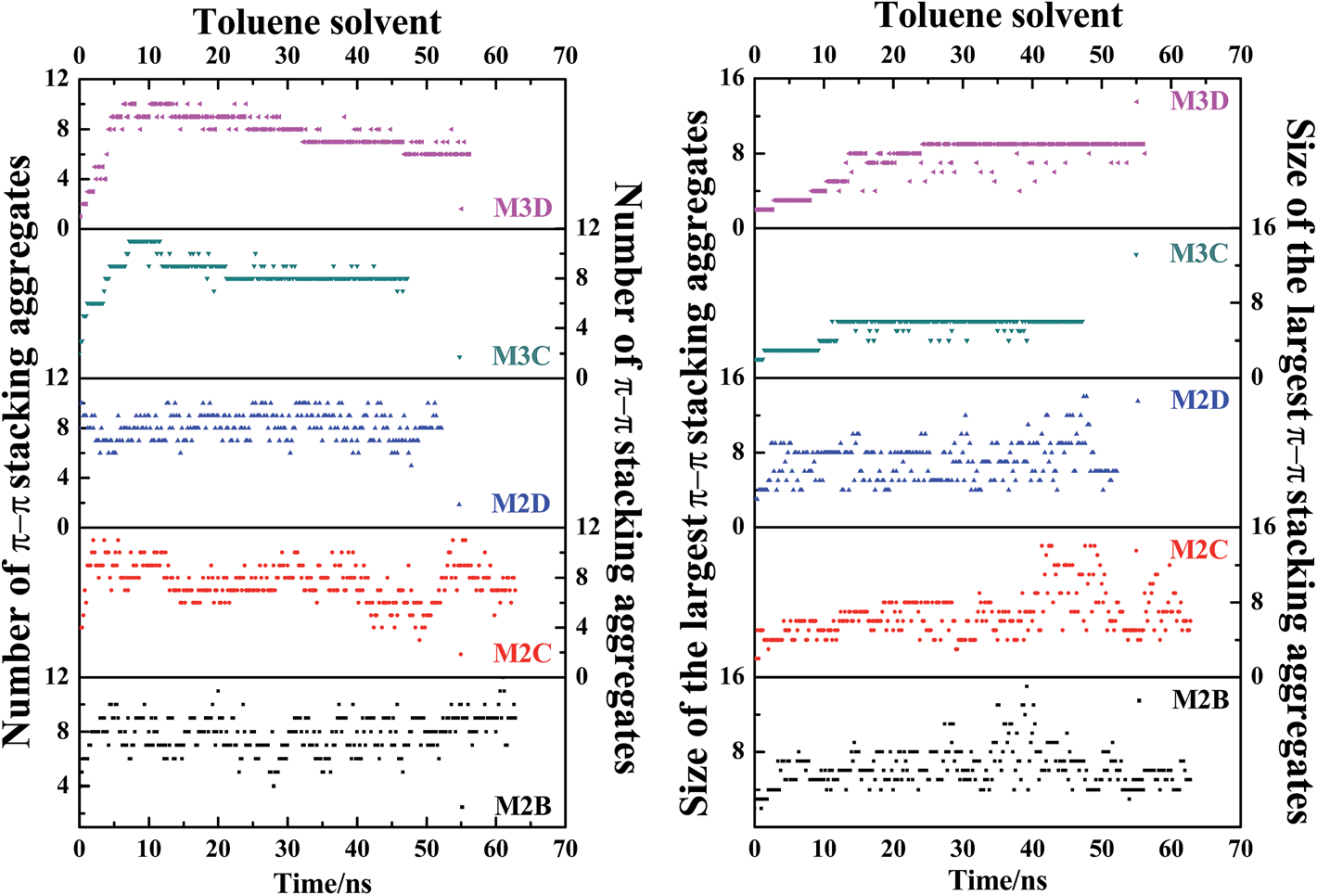
The evolution of size and number of π–π stacking molecular aggregates in five systems (M2B, M2C, M2D, M3C, M3D dissolved in excess toluene solvent) along with the time of MD simulations. The size of the largest π–π stacking molecular aggregate is defined by the number of asphaltene molecules within the largest-sized aggregate. The molecular snapshots of nanoaggregates at different simulation stages are displayed in Fig. S10.†

In order to track the dynamic process of deformation and reformation of molecules in nanoaggregates, we counted the size and number of nanoaggregates at different simulation stages. From Fig. 8, the nanoaggregates formed by five types of model asphaltene molecules in the toluene solvent show much different dynamic properties caused by the side chain configurations. The number and size of nanoaggregates formed by the M2B, M2C, and M2D molecules fluctuate much more rapidly in comparison to that of the M3C and M3D nanoaggregates, which indicates that the nanoaggregates formed by the M2B, M2C, and M2D molecules undergo rapid deformation and reformation. As for the M3C and M3D molecular systems, as shown in Fig. 8, the number and size of the nanoaggregates are quite stable after reaching the equilibrium state.

**Fig. 9:**
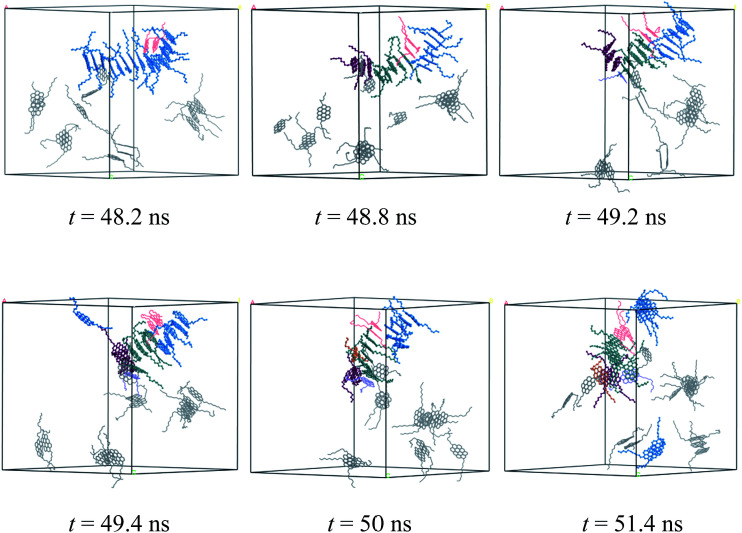
Atomic details of the deformation and reformation of nanoaggregates formed by the M2D molecules in the toluene solvent. The cell parameters are *a* = *b* = *c* = 84.78 Å.

The dynamic process of deformation and reformation of nanoaggregates is probed. Because the nanoaggregates formed by M2B, M2C, and M2D molecules show much rapid deformation and reformation, here, we take the M2D system as an example to explore the possible deformation and reformation mechanism of nanoaggregation. As shown in Fig. 8, a rapid deformation-reformation process of nanoaggregation is found at approximately 50 ns simulation in the M2D system. Fig. 9 illustrates this process with atomic details. From Fig. 9, at *t* = 48.2 ns, a large-sized nanoaggregate containing 14 asphaltene molecules is seen. At the side of this large-sized nanoaggregate, there is a T-shaped packing asphaltene molecular dimer. When the asphaltene molecular dimer approaches the large-sized nanoaggregate, it can attach to the asphaltene molecules in the middle part of the larger-sized nanoaggregate (*t* = 48.2 ns) to form new π–π stacking structures. At this moment, the large-sized nanoaggregate deformed into three smaller-sized nanoaggregates containing 6, 7, and 3 asphaltene molecules with T-shape stacking orientations. These newly formed smaller nanoaggregates may further diffuse in the toluene solvent and interact with other nanoaggregates to form new aggregates. We call this process the nanoaggregate-induced deformation and reformation process. The π–π interactions of asphaltene molecules in nearby nanoaggregates are the major driving force that induces the deformation and reformation of asphaltene nanoaggregate.

## 4. Conclusions

Atomistic MD simulations were employed to study the organizational behavior of five model alkyl side chain-decorated ‘continental’ type asphaltene molecules containing a polycyclic aromatic nucleus. By adjusting the position, number, and length of the alkyl side chains surrounding the aromatic nucleus, the side chain effects of the molecular organizational structure without toluene molecules or in the presence of toluene additives and toluene solvent were discussed. The symmetrical decoration of two alkyl side chains on the condensed aromatic cores lead to a novel ‘phase separated lamellar structure’ (PSLS). A ‘phase separated columnar structure’ (PSCS) is observed for the ‘continental’ type asphaltene with evenly distributed highly branched alkyl side chains. The formation of PSLS and PSCS is also found in the presence of a small amount of toluene additives (30% wt fraction). The toluene molecules are primarily distributed in the region of alkyl side chains. The driving force to form such types of PSLS and PSCS is considered to be π–π interactions. We also investigated the effects of side chains on the packing structure and dynamic behaviors of asphaltene nanoaggregates in the toluene solvent. It was found that the polyaromatic molecules with highly branched side chains formed larger-sized and more stable nanoaggregates than the polyaromatic molecules decorated with two alkyl side chains. Furthermore, a nanoaggregate-induced deformation and reformation mechanism is suggested.

## Conflicts of interest

The authors declare no competing financial interests.

## Supplementary Material

RA-008-C7RA13101A-s001
