# Supplementary material for: Molecular dynamics simulations of the self-organization of side-chain decorated polyaromatic conjugation molecules: phase separated lamellar and columnar structures and dispersion behaviors in toluene solvent
Source: RSC Adv. 2018 Mar 21;8(20):11134–44. doi: 10.1039/c7ra13101a (PMC9078946; doi:10.1039/c7ra13101a)
Supplement: RA-008-C7RA13101A-s001 [file RA-008-C7RA13101A-s001.pdf]

Electronic Supplementary Material (ESI) for RSC Advances.
This journal is © The Royal Society of Chemistry 2018

Supplementary Information

Molecular Dynamics Simulations of the Self-Organization of Side-Chain Decorated Polyaromatic Conjugation Molecules: Phase Separated Lamellar and Columnar Structures and Dispersion Behaviors in Toluene Solvent

Lanyan He[a], Pingmei Wang[c,d], Lipeng He[c,d], Zhou Qu[a], Jianhui Luo[c,d]*, Baoliang Peng[c,d], Xianqiong Tang[b]*, Yong Pei[a]*

[a]*Key Laboratory for Green Organic Synthesis and Application of Hunan Province ,Key Laboratory of Environmentally Friendly Chemistry and Applications of Ministry of Education, Xiangtan University, Hunan Province 411105, China*

[b]*Department of Civil Engineering and Mechanics,Xiangtan University, Xiangtan 411105, China*

[c]*Research Institute of Petroleum Exploration & Development (RIPED), PetroChina, Beijing 100083, China.*

[d]*Key Laboratory of Nano Chemistry (KLNC), CNPC, Beijing 100083, China*

The time required to obtain a stable PSLS from an initial, completely random structure is different for **M2B、M2C and M2D** system containing two symmetric alkyl side chains, as shown in **Figure S1-S3**.

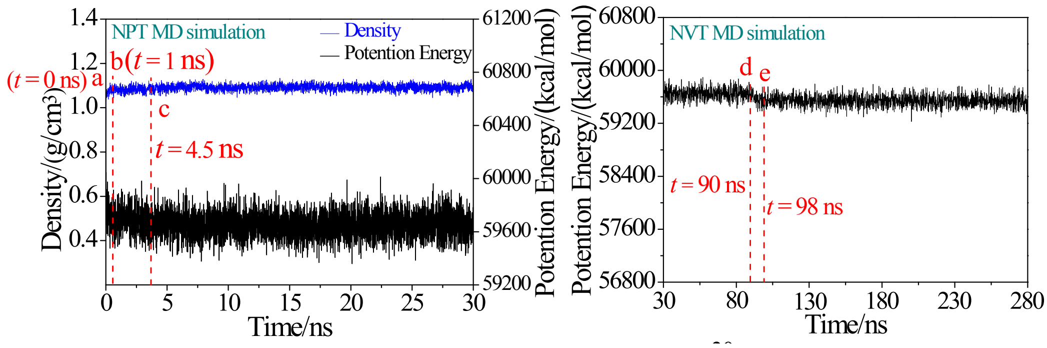

(I) **(II)**

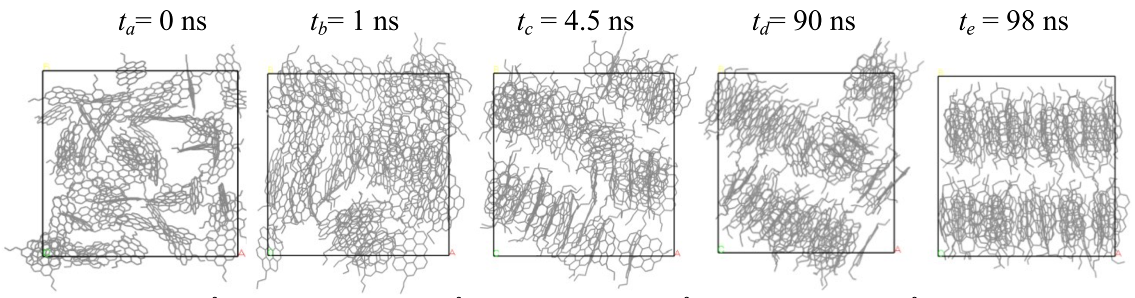

a=b=c= 43.11 Å a=b=c= 35.31 Å a=b=c= 35.39 Å a=b=c= 35.29 Å a=b=c= 35.29 Å

(III)

Figure S1 Evolution of density and potential energy of **M2B** molecular system during **(I)** NPT MD simulation (30 ns) and **(II)** subsequent NVT MD simulation (250 ns). **(III)** The snapshots of trajectory at different simulation time. H atoms were hidden for clarity.

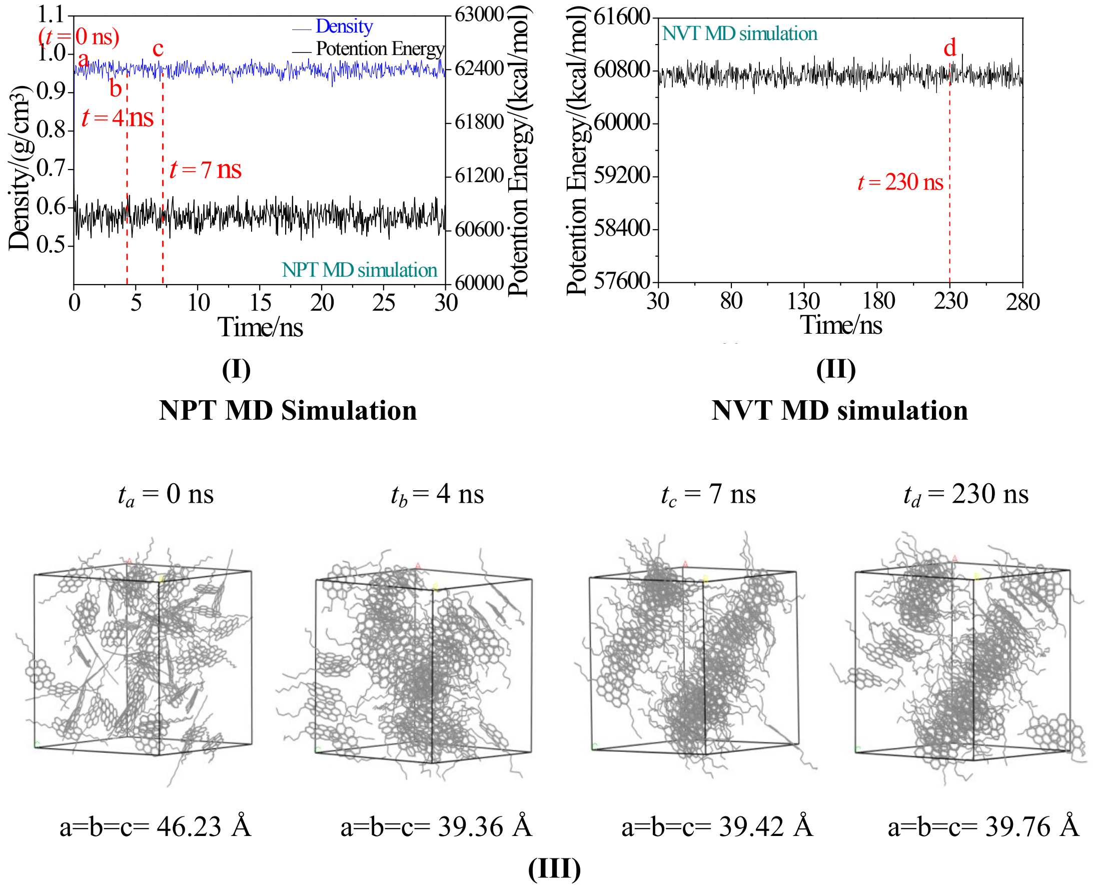

Figure S2 Evolution of density and potential energy of **M2C** molecular system during **(I)** NPT MD simulation (30 ns) and **(II)** subsequent NVT MD simulation (250 ns). **(III)** The snapshots of trajectory at different simulation time. H atoms were hidden for clarity.

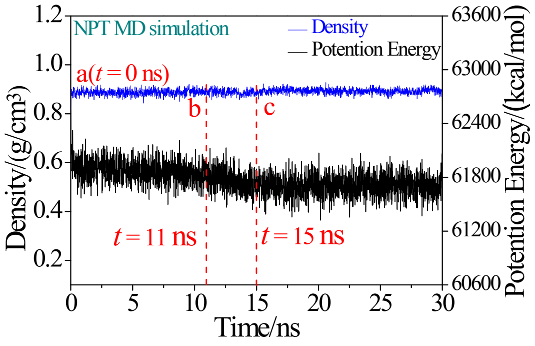

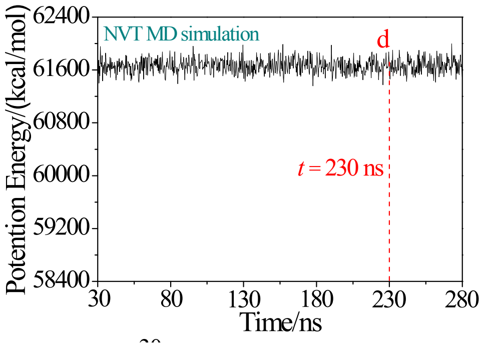

(I)

NPT MD Simulation

(II)

NVT MD simulation

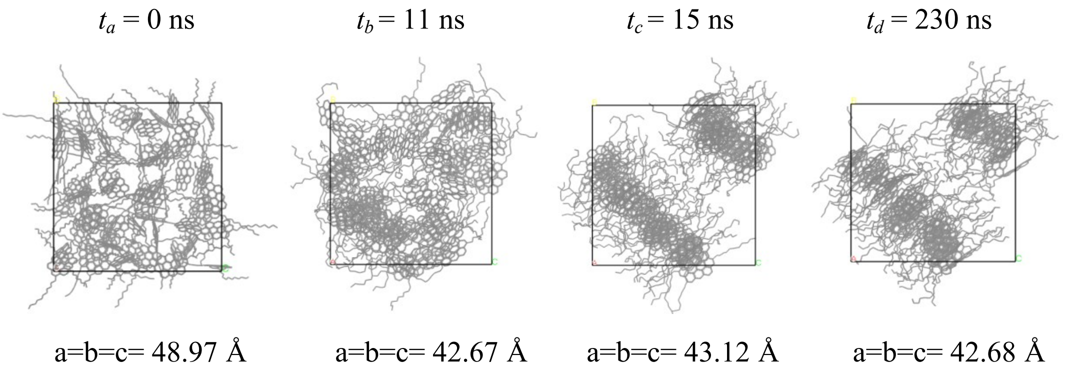

(III)

Figure S3 Evolution of density and potential energy of **M2D** molecular system during **(I)** NPT MD simulation (30 ns) and **(II)** subsequent NVT MD simulation (250 ns). **(III)** The snapshots of trajectory at different simulation time. H atoms were hidden for clarity.

The evolution of density and potential energy and formation process of ordered ‘columnar’ stacking structures of **M3C** and **M3D** molecular systems are summarized in **Figure S4 and S5**.

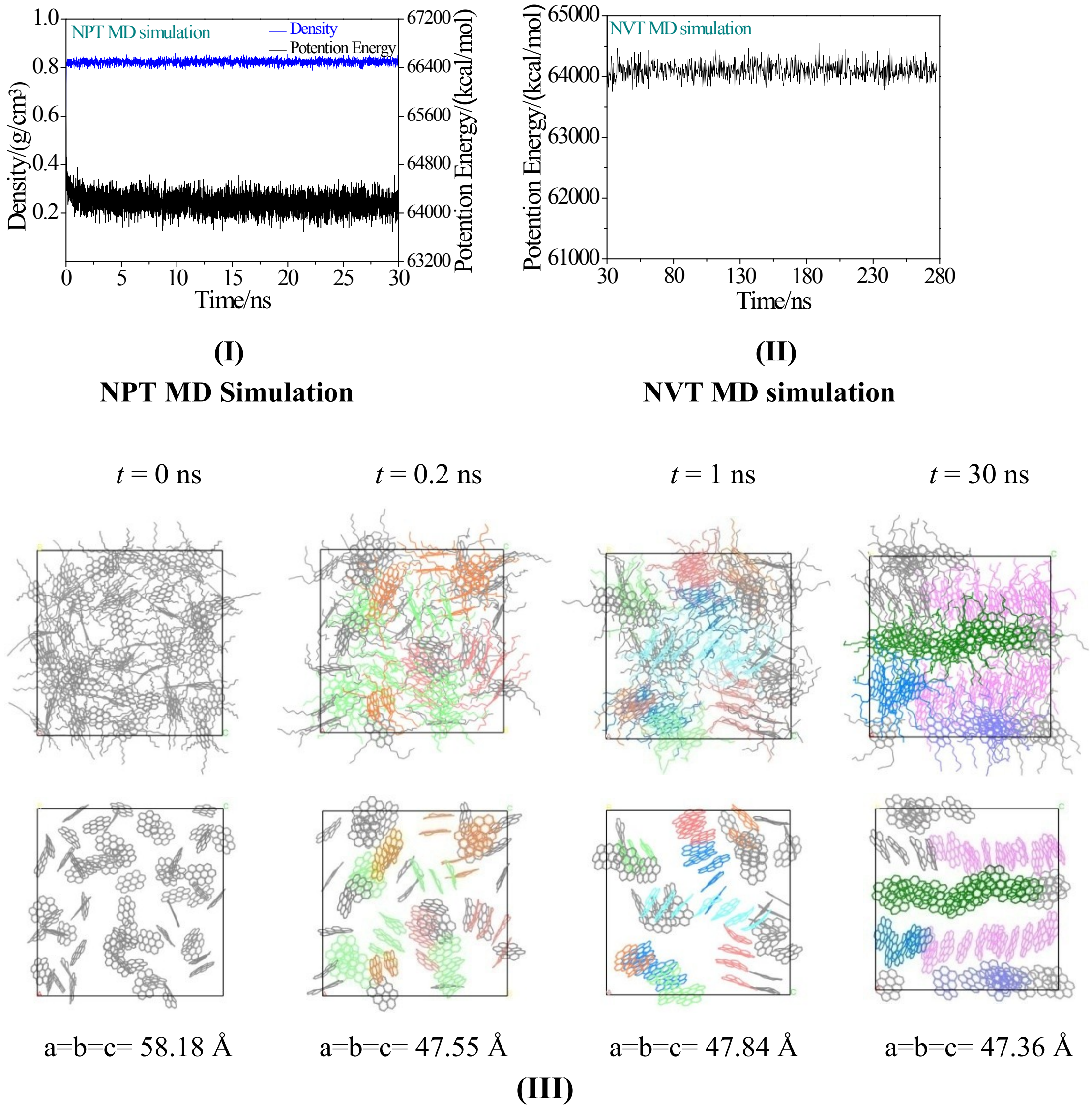

Figure S4 Evolution of density and potential energy of **M3C** molecular system during **(I)** NPT MD simulation (30 ns) and **(II)** subsequent NVT MD simulation (250 ns). **(III)** The snapshots of trajectory at different simulation time, and the four snapshots below hidden the side chains to see more clearly the changes in aggregates in systems. The molecular dimer, trimer, tetramer, pentamer, hexamer and heptamer aggregation structures are colored by orange, green, red, blue, purple, and sky-blue, respectively. H atoms were hidden for clarity.

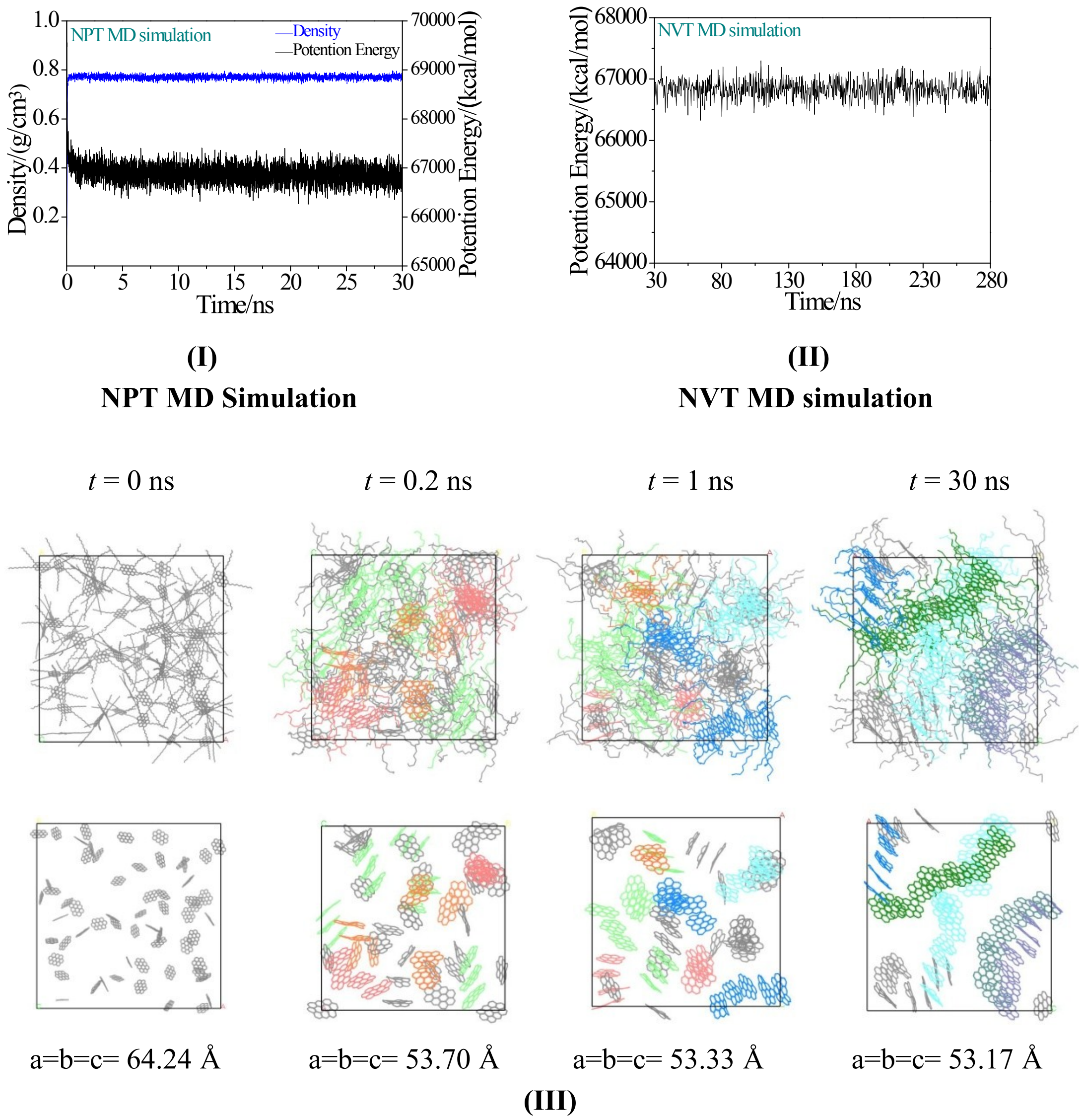

Figure S5 Evolution of density and potential energy of **M3D** molecular system during **(I)** NPT MD simulation (30 ns) and **(II)** subsequent NVT MD simulation (250 ns). **(III)** The snapshots of trajectory at different simulation time, and the four snapshots below hidden the side chains to see more clearly the changes in aggregates in systems. The molecular dimer, trimer, tetramer, pentamer and heptamer aggregation structures are colored by orange, green, red, blue and sky-blue, respectively. H atoms were hidden for clarity.

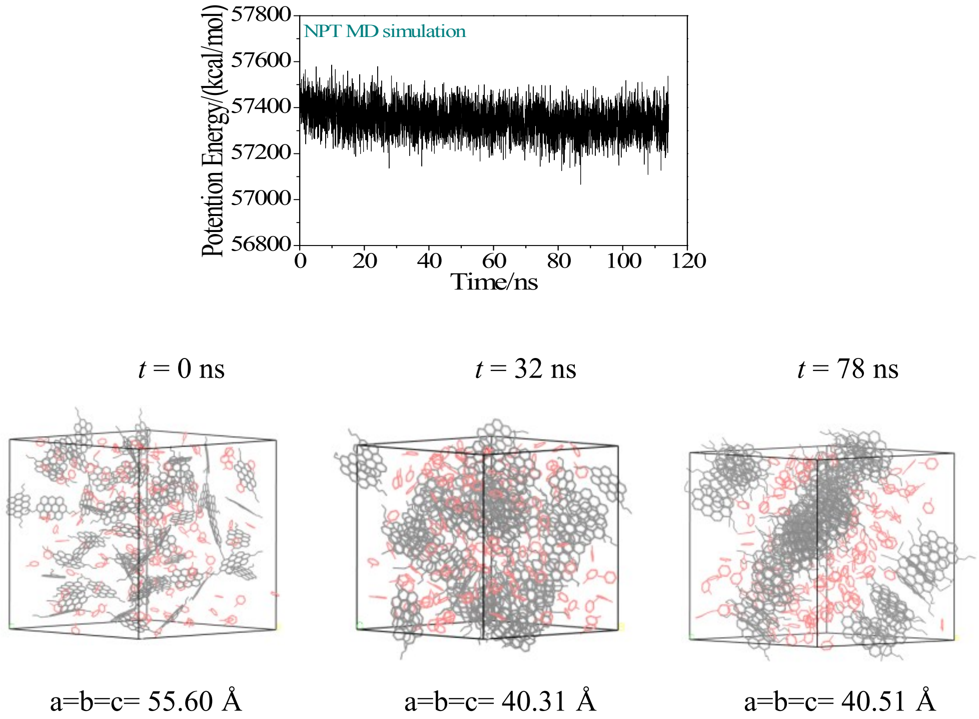

(a) M2B + 30 wt% toluene

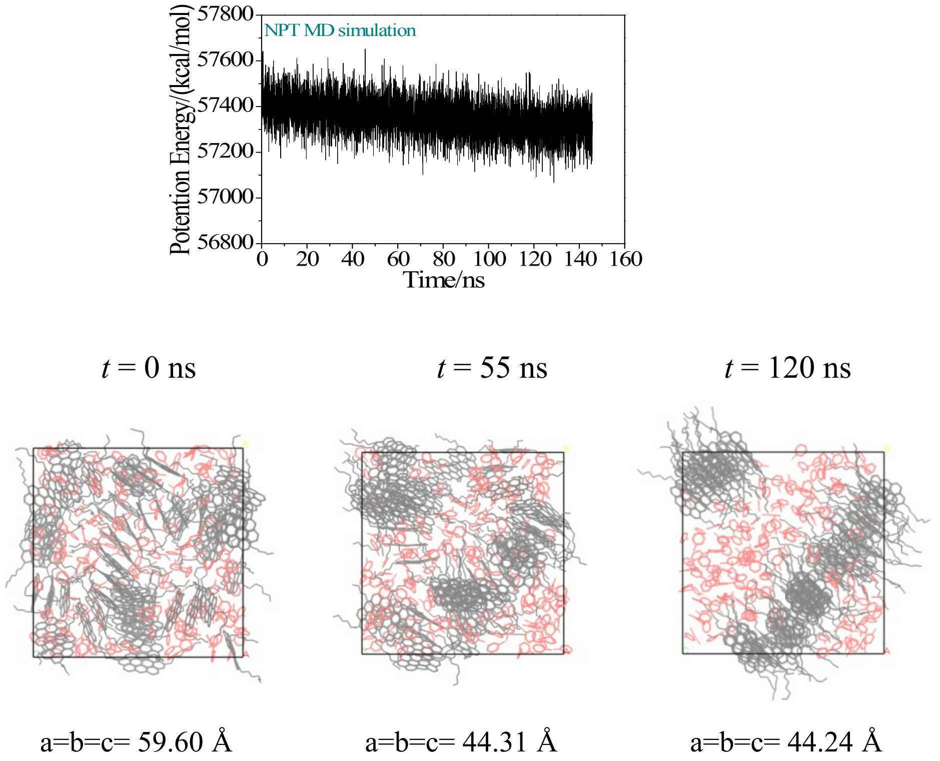

(b) M2C + 30 wt% toluene

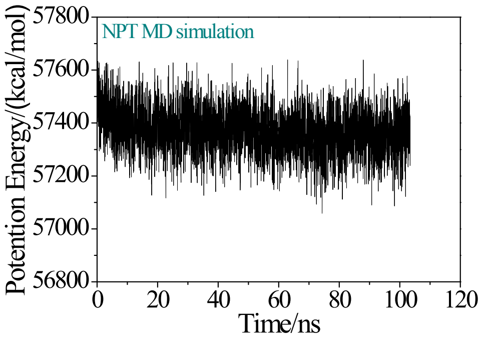

t = 0 ns   *t* = 70 ns   *t* = 100 ns

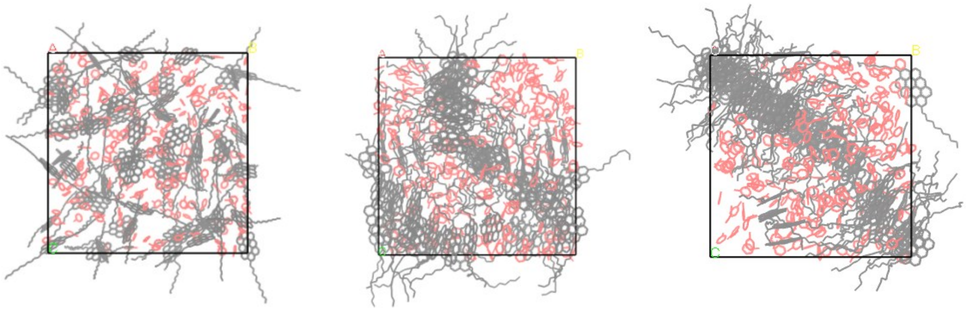

a=b=c= 63.11 Å   a=b=c= 47.46 Å   a=b=c= 47.51 Å

(c) M2D + 30 wt% toluene

Figure S6 Evolution of potential energy and the snapshots of trajectory at different simulation time of mix asphaltene ((a) **M2B**, (b) **M2C**, and (c) **M2D**) and toluene solvent (30% wt.) molecular systems plus. The H atoms are hidden for clarity.

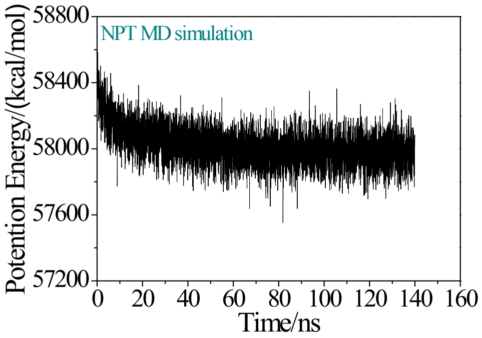

t = 0 ns | t = 6 ns | t = 110 ns

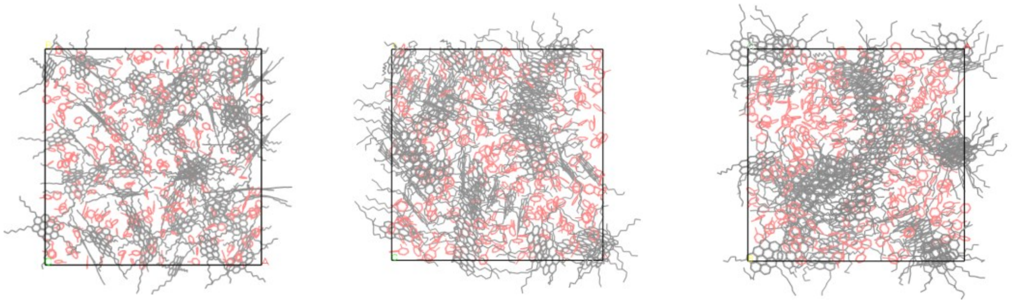

a=b=c= 68.14 Å | a=b=c= 52.06 Å | a=b=c= 51.94 Å

(a) M3C + 30 wt% toluene

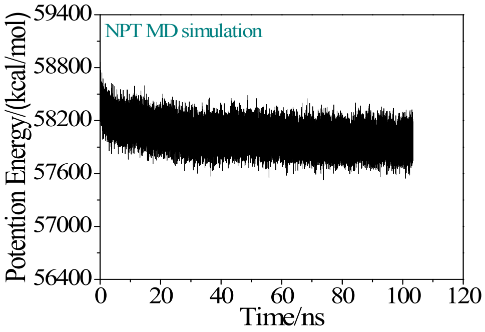

(I)

t = 0 ns | t = 2 ns | t = 80 ns

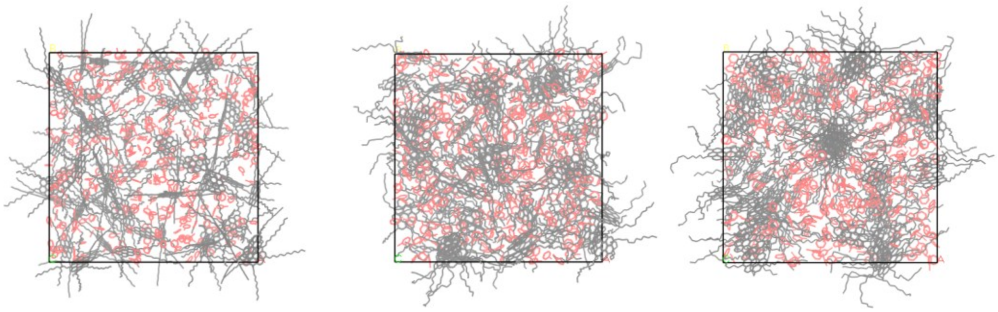

a=b=c= 74.66 Å | a=b=c= 57.81 Å | a=b=c= 57.72 Å

(a) M3D + 30 wt% toluene

Figure S7 Evolution of potential energy and the snapshots of trajectory at different simulation time of mix asphaltene ((a) **M3C**, (b) **M3D**) and toluene solvent (30% wt.)

molecular systems plus. The H atoms are hidden for clarity.

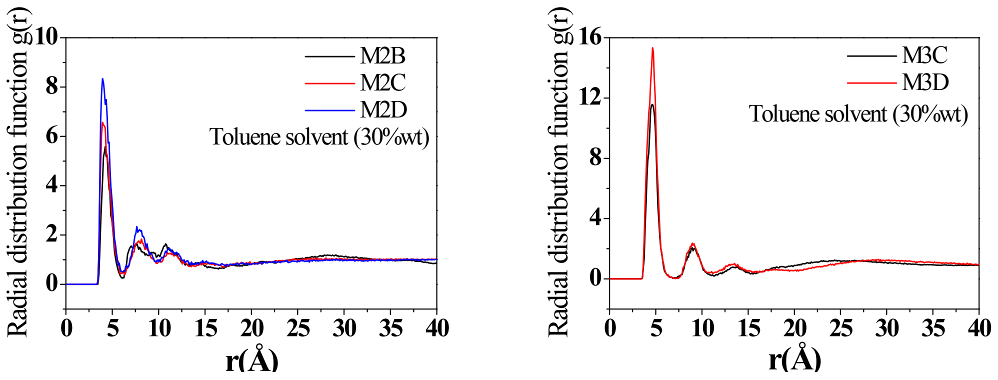

Figure S8 The radical distribution function g(*r*) of asphaltene-asphatene molecules in five molecular aggregation structures (*r* is the distance between the centers of aromatic cores). 30 % weight toluene solvent is mixed with the asphaltene molecules.

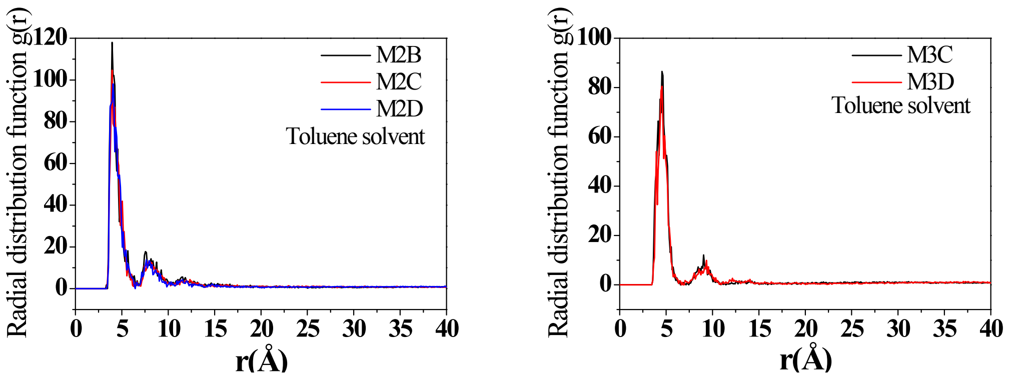

Figure S9 The radical distribution function g(*r*) of asphaltene-asphatene molecules in five molecular aggregation structures (*r* is the distance between the centers of aromatic cores). Asphaltene molecules are dissolved in excess toluene solvent.

t = 5 ns | *t* = 25 ns | *t* = 60 ns

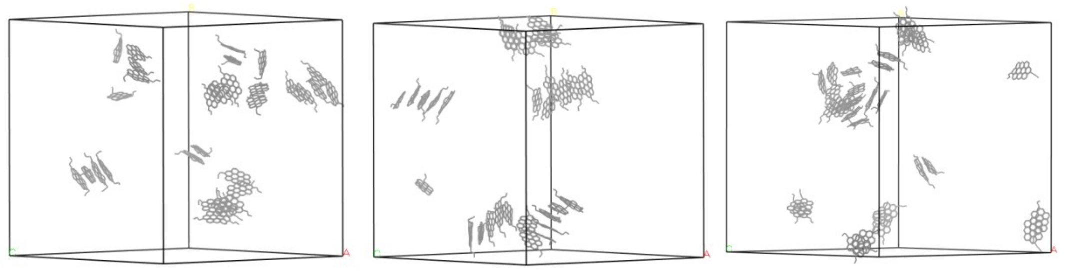

a=b=c= 84.30 Å | a=b=c= 84.21 Å | a=b=c= 84.17 Å

(a) Nanoaggregates of M2B in toluene solvent

t = 5 ns | *t* = 25 ns | *t* = 67 ns

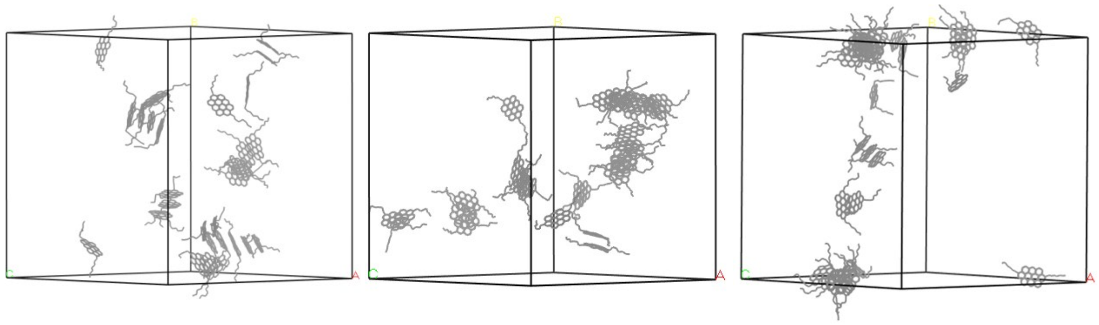

a=b=c= 84.37 Å | a=b=c= 84.64 Å | a=b=c= 86.05 Å

(b) Nanoaggregates of M2C in toluene solvent

t = 5 ns | *t* = 25 ns | *t* = 55 ns

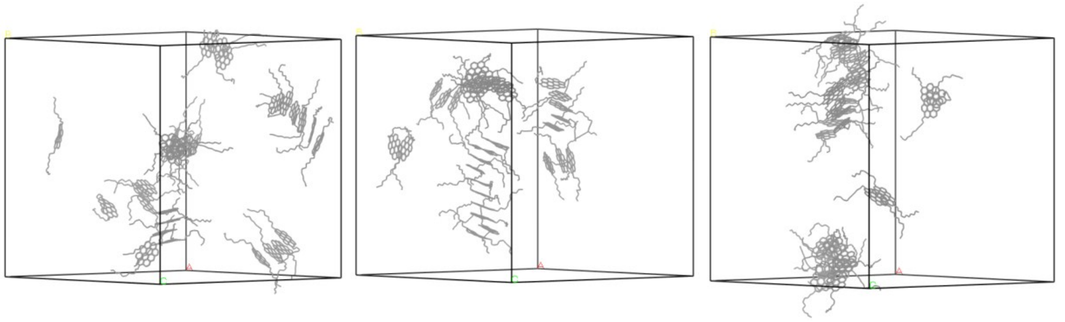

a=b=c= 84.72 Å | a=b=c= 84.85 Å | a=b=c= 84.88 Å

(c) Nanoaggregates of M2D in toluene solvent

t = 5 ns *t* = 25 ns *t* = 40 ns

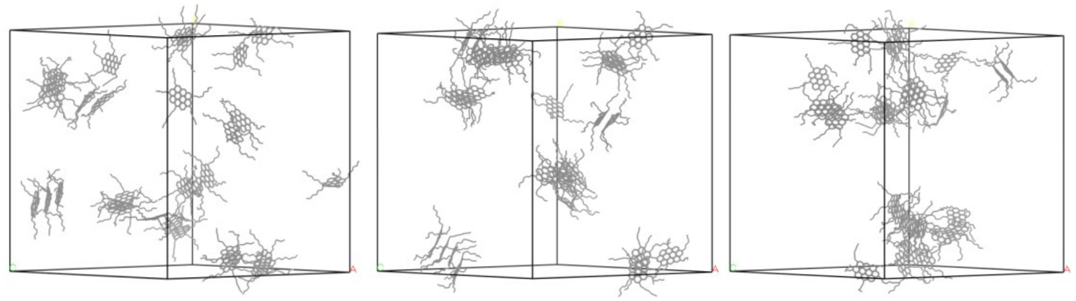

a=b=c= 85.37 Å a=b=c= 85.33 Å a=b=c= 85.53 Å

(d) Nanoaggregates of M3C in toluene solvent

t = 5 ns *t* = 25 ns *t* = 50 ns

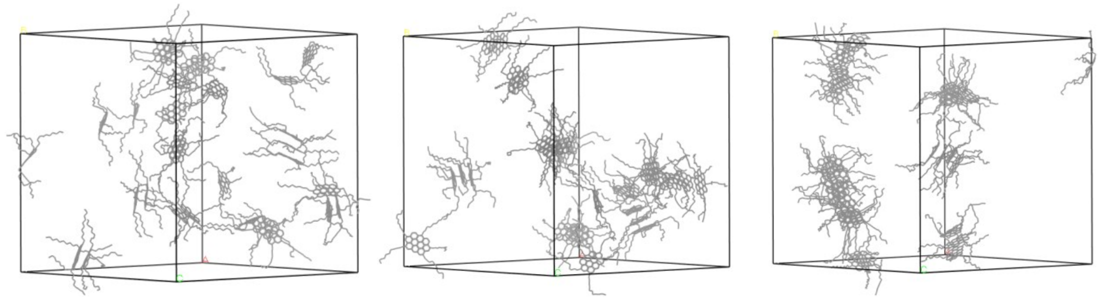

a=b=c= 86.09 Å a=b=c= 86.21 Å a=b=c= 86.20 Å

(e) Nanoaggregates of M3D in toluene solvent

Figure S10 Molecular snapshots of nanoaggregates of five kinds of model asphaltene molecules at different stage of MD simulations. Toluene solvents are not displayed.
